# HPLC-MS/MS and ICP-MS for Evaluation of Mycotoxins and Heavy Metals in Edible Insects and Their Defatted Cakes Resulting from Supercritical Fluid Extraction

**DOI:** 10.3390/foods13203233

**Published:** 2024-10-11

**Authors:** Lucia Cuesta Ramos, Aroa Rodríguez-García, Juan M. Castagnini, Manuel Salgado-Ramos, Pedro V. Martínez-Culebras, Francisco J. Barba, Noelia Pallarés

**Affiliations:** 1 Research Group in Innovative Technologies for Sustainable Food (ALISOST), Department of Preventive Medicine and Public Health, Food Science, Toxicology and Forensic Medicine, Faculty of Pharmacy, Universitat de València, Avda. Vicent Andrés Estellés, s/n, 46100 Burjassot, Valencia, Spainrogara8@alumni.uv.es (A.R.-G.); juan.castagnini@uv.es (J.M.C.); manuel.salgado@uclm.es (M.S.-R.); francisco.barba@uv.es (F.J.B.); noelia.pallares@uv.es (N.P.); 2 Facultad de Ciencias y Tecnologías Químicas, Universidad de Castilla-La Mancha, Avda. Camilo José Cela 10, 13005 Ciudad Real, Castile-La Mancha, Spain

**Keywords:** edible insects, mycotoxins, heavy metals, SFE extraction, ICP-MS, UHPLC-MS/MS

## Abstract

Edible Insects (EIs) are an alternative source of bioactive compounds such as proteins or fatty acids and micronutrients as vitamins or minerals, thus showing potential to replace traditional foodstuffs in an economical and environmentally friendly way. Nonetheless, EIs can accumulate hazardous chemicals such as mycotoxins and heavy metals. The aim of the present study is to determine mycotoxins and heavy metal content in raw insect samples and those resulting products obtained after supercritical fluid extraction (SFE). Insect samples included *Acheta domesticus* (cricket) meal, *Tenebrio molitor* (mealworm) meal, *Alphitobius diaperinus* (buffalo worm), and *Locusta migratoria* (locust). For this purpose, a QuEChERS method followed by LC-MS/MS analysis was optimized with good results for the analysis of mycotoxins, principally Aflatoxins (AFs), Ochratoxin A (OTA), and Enniatins (ENNs). In contrast, heavy metals (As, Cd, Hg, Pb) were determined by Inductively Coupled Plasma Mass Spectrometry (ICP-MS). The results obtained revealed that Locust was positive for AFG2 at a level of 115.5 μg/kg, and mealworm was only contaminated with OTA at 58.1 μg/kg. Emerging mycotoxins (ENNA, ENNA1, ENNB, and ENNB1) were detected at lower levels < 2.2 µg/Kg. Concerning heavy metals, limits exceeding regulation were detected for Cd in the insect species studied, with levels up to 219 μg/kg, and for Pb in crickets (100.3 μg/kg). Finally, the analysis of the post-extraction solids after SFE processing revealed that heavy metals remained in the resulting SFE cakes, while mycotoxins were detected at negligible levels (up to 1.3 µg/Kg).

## 1. Introduction

The United Nations (UN) Food Systems Summit, held during the UN General Assembly on 23 September, recognized the need to take action to bring about tangible and positive change in the world’s food systems to achieve the Sustainable Development Goals by 2030 (United Nations, 2023).

In recent years, there has been a growing public concern about the environmental impact of extensive livestock farming, as a result of which alternative environmentally friendly sources of protein are emerging [1,2]. For instance, proteins derived from plants, insects, algae, bacteria, or fungi are noticeable [3,4]. Moreover, these novel sources are generally inexpensive and highly nutritious [5]. The advantages of these alternative protein sources have led to their research and incorporation into various food formulations [6].

The nutritional content of Edible Insects (EIs) varies according to species, growth stage, and feed, but, in general, they are rich in protein, including essential amino acids, with protein digestibility values similar (or slightly lower) to egg or beef protein [7]. Moreover, EIs contain monounsaturated fatty acids (MUFAs) and/or polyunsaturated fatty acids (PUFAs). Their presence in several micronutrients, such as vitamins (riboflavin, pantothenic acid, biotin, and, in some cases, folic acid) and minerals (iron, magnesium, manganese, phosphorus, copper, zinc, and selenium), is also noteworthy [8].

Farmed insects should meet food safety requirements and provide solutions in terms of production costs, sustainability, scalability, and consumer acceptance [9,10]. The insect production sector offers opportunities for a circular and environmentally friendly approach. Insect cultivation results in approximately 40–60% lower land use, water footprint, ammonia, and greenhouse gas emissions in comparison to traditional animal livestock [11]. Currently, four insect species are already authorized by Regulation (EU) (2015/2283): the larvae of the mealworm (*Tenebrio molitor*), the migratory locust (*Locusta migratoria*), the house cricket (*Acheta domesticus*), and the larvae of the dung beetle (*Alphitobius diaperinus*).

Although using insects as a food source has important benefits, insects, like other animals, can accumulate harmful microorganisms and hazardous chemicals, including heavy metals, mycotoxins, dioxins, pesticide residues, and veterinary drugs [12]. Some factors such as insect species, harvesting stage, production conditions, and the feeding substrate can influence the occurrence and accumulation of contaminants. Thus, insects tend to be contaminated by the substrate used for their breeding and development or by improper processing and storage operations. For this reason, farmed insects should be routinely monitored for the presence of chemical contaminants [13,14].

Mycotoxins are toxic chemical compounds produced in the food chain by filamentous fungi, mainly species from *Aspergillus*, *Penicillium*, and *Fusarium* genera, through the infection of crops both before and after harvest [15]. Mycotoxins are a major food safety concern due to their severe toxic and carcinogenic effects on humans and animals [16]. More than 300 different mycotoxins have been identified, which include ochratoxins (OTs), aflatoxins (AFs), deoxynivalenol (DON), and zearalenone (ZEN) as the most relevant in food crops. All of them are regulated after a thorough risk assessment procedure [17]. In addition, emerging mycotoxins are attracting increasing interest among the scientific community, such as the *Fusarium* mycotoxins enniatins (ENNs) [18]. Consumption of major mycotoxin-containing food or feed may cause adverse health effects in humans or animals, which induce hepatotoxicity, immunotoxicity, membrane damage, gastrointestinal toxicity, cardiotoxicity, nephrotoxicity, pulmonary toxicity, neurotoxicity, genotoxicity, teratogenicity, and sometimes lead to cancer development [19,20].

Heavy metals are accumulating in the environment, becoming a big food safety concern. These compounds are not metabolized into other intermediates, making them difficult to break down in the body [21]. The presence of heavy metals such as arsenic, mercury, cadmium, and lead has been reported in EIs. The accumulation of heavy metals in insects is mainly associated with agricultural wastes used to feed insects [22]. When heavy metals enter the food chain, they induce cytotoxicity through molecular mechanisms, including the production of reactive oxygen species inducing protein modification, lipid peroxidation, or DNA damage, among others, which cause adverse effects such as various types of cancer, neurological and cardiovascular disorders, damage to renal function, and other endocrine abnormalities [23].

Furthermore, the available literature has shown that heavy metals can accumulate in insects, while mycotoxins can be partially degraded by insects, although the metabolic pathways involved need to be further investigated [24]. Further information on the occurrence of heavy metals and mycotoxins in EIs is, therefore, warranted.

In trying to implement sustainable practices focusing on mitigating contaminants from foods, supercritical fluid extraction (SFE) arises as an environmentally friendly, cost-effective extraction method that allows the recovery of extracts with high extraction efficiency and selectivity. SFE uses solvents at their supercritical temperature and pressure point to recover solutes from solid matrices under pressurized conditions. Under these conditions, solvents exhibit properties intermediate between liquids and gases, resulting in a higher diffusion coefficient and lower viscosity, which enhances solvent penetration into the matrix. As a result of SFE, valuable bioactive compounds, principally lipids but also polyphenols or fat-soluble vitamins, are extracted. Therefore, SFE has a promising potential in food processing applications [25,26,27].

The aim of this study is to determine the presence of mycotoxins and heavy metals in EIs. For this purpose, a QuEChERS method followed by LC-MS/MS analysis was optimized for the analysis of mycotoxins, with heavy metals evaluated by ICP-MS. In addition, insect samples were extracted using supercritical fluid extraction (SFE) technology to obtain the remaining cakes rich in proteins. Finally, the defatted cakes were analyzed for the presence of mycotoxins and heavy metals.

## 2. Materials and Methods

### 2.1. Reagents and Chemicals

Deionized water (resistivity > 18 MΩ/cm) was obtained using a Milli-Q SP^®^ water system (Millipore Corporation, Bedford, MA, USA). Ammonium formate (99%) was supplied by Panreac Química S.A.U. (Barcelona, Spain). Formic acid (reagent grade ≥ 95%) was provided by Sigma-Aldrich (St. Louis, MO, USA). Acetonitrile (ACN). Ethanol and methanol (MeOH) (HPLC grade) were purchased from Merck (Darmstadt, Germany). All solvents were filtered through a 0.45 μm cellulose filter from Scharlau (Barcelona, Spain) before use. Sodium chloride (NaCl) was obtained from VWR Chemicals (Leuven, Belgium), magnesium sulfate (MgSO_4_) anhydrous 99.5% was supplied by Alfa Aesar (Karlsruhe, Germany), and octadecyl C18 sorbent was purchased from Phenomenex (Madrid, Spain). Before injection, samples were filtered through a nylon filter (13 mm/0.22 μm) from Membrane Solutions (Plano, TX, USA). Heavy metal solution standards and nitric acid (HNO_3_) were purchased from Merck (Darmstadt, Germany). Mycotoxin standards (ochratoxin A (OTA), aflatoxin B1 (AFB1), aflatoxin B2 (AFB2), aflatoxin G1 (AFG1), aflatoxin G2 (AFG2), enniatin A (ENNA), enniatin A1 (ENNA1), enniatin B (ENNB), and enniatin B1 (ENNB1)) were supplied by Sigma (St. Louis, MO, USA). Individual stock solutions of each mycotoxin were prepared in MeOH at a concentration of 100 mg/L. Working solutions were prepared from these individual stock solutions. All prepared solutions were stored in the dark at −20 °C until analysis.

### 2.2. Samples

Insects for human consumption were purchased from a local market in Valencia (Spain). They included *Acheta domesticus* (cricket) flour, *Tenebrio molitor* (mealworm) flour, *Alphitobius diaperinus* (buffalo worm), and *Locusta migratoria* (locust). Whole insects were ground in the laboratory using a Moulinex^®^ before analysis.

### 2.3. Supercritical Fluid Extraction (SFE)

Supercritical fluid extraction (SFE) was performed in a JASCO (Tokyo, Japan) system consisting of an isocratic CO_2_ pump (PU-4387) with a flow range of 5 to 40 mL/min, adjustable in 0.01 mL/min increments, with a maximum pressure of 50 MPa and a pulse extraction system. It also included a cooling circulator (JULABO FL 1201) for cooling the pump in the temperature range −20–40 °C, with a flow range of up to 23 L/min and a cooling capacity of 1.2 kW at 20 °C. It includes another isocratic organic modifier pump (PU-4086, HPLC) with a flow range of 0.001 to 10 mL/min, adjustable in 0.01 mL/min increments and a maximum pressure set to 70 MPa, and a thermostatic glass reaction oven (CO-4065) with a temperature range of 4 to 90 °C. It also includes a thermostatic system with Peltier and airflow temperature transfer, a pressure regulator (BP-4340), and a 5 mL supercritical extraction vessel (803559—5 mL).

For extraction, 3 g of each insect was charged in the reactor. The extraction parameters were set based on the work reported by Laroche et al. [28]: 75 min, 55 °C, and 32.5 MPa. Extraction was assisted by supercritical CO_2_ with a low proportion of modifying polar phase (EtOH) (CO_2_: ethanol 92:8 ratio). The flow rate was constant at 10 mL/min.

After extraction, oily extracts and residual cakes were obtained from the raw insects and stored at 4 °C until analysis. Experiments were performed in duplicate.

### 2.4. Mycotoxins Extraction

The analysis was performed using a QuEChERS method for mycotoxin extraction, which was validated in-house. A total of 2 g of the powdered insect sample was weighed into a 50 mL Falcon tube. This was followed by the addition of 10 mL of 2% formic acid water and agitation for 30 min at 5 Hz on a KS 260 IKA shaker (Staufen, Germany). Subsequently, 10 mL of acetonitrile (ACN) was added to the tube and shaken again for 30 min more on the same shaker. Then, 4 g of MgSO_4_ and 1 g of NaCl were added and vortexed for 30 s. The tubes were centrifuged in an Eppendorf Centrifuge 5810R at 50 Hz for 10 min (Madrid, Spain). After centrifugation, 2 mL of the supernatant was removed and transferred to a 15 mL Falcon tube. Then, 0.3 g of MgSO_4_ and 0.1 g of octadecyl C18 were added, vortexed for 30 s, and centrifuged at 50 Hz for 10 min. Finally, the supernatant obtained was filtered through a 13 mm/0.22 μm nylon filter (Membrane Solutions, Plano, TX, USA). The resulting vials were introduced into the UHPLC-MS/MS system.

### 2.5. UHPLC-MS/MS Analysis

The determination of mycotoxins (AFB1, AFB2, AFG1, AFG2, OTA, ENNA, ENNA1, ENNB, ENNB1) was performed by UHPLC-MS/MS using a Sciex TRIPLE QUAD 6500+ equipped with electrospray ionization (ESI) coupled to an Agilent 1260 HPLC UHPLC system (degasser), quaternary pump and column oven with an Eksigent ULC 100 HTC-xt autosampler. A BEH^®^ C18 column (1.7 μm; 100 Å, LC 50 × 2.1 mm column, Waters) was used. The mobile phases were (A) 5 mM ammonium formate and 0.1% formic acid H_2_O and (B) methanol (0.1% formic acid). The linear gradient used was 0 min (95% A), 2 min (95% A), 13 min (0% A), 15.1 min (5% A), and 18 min (5% A) with an injection volume of 5 μL and a constant temperature of 30 °C on the column. The mass spectrometer was operated in positive ionization mode and Selected Reaction Multiple Monitoring (SRM) mode using a Turbo Spray IonDrive ionization source with the following conditions: curtain gas (CUR) 30 PSI, ion sputtering voltage (IS) 4.4 kV, temperature 300 °C, and ion source gas (GS) 1 and 2 at 55 psi. The UHPLC-MS/MS conditions and the method performance parameters are given in Table 1.

### 2.6. Method Optimization

The method was optimized for insect samples in terms of matrix effects (signal suppression enhancer), recoveries, repeatability (intra-day precision), reproducibility (inter-day precision), linearity, and limits of detection (LODs) and quantification (LOQs) according to Commission Decision (2002/657/EC) (Table 1) [29].

To determine linearity, calibration curves were performed for each mycotoxin studied, dissolved in methanol at concentrations ranging from the LOQ to 500 μg/L, and calibration curves were performed in a blank extract sample (in this case, an extract from a mycotoxin-free cricket sample). All mycotoxins analyzed showed good linearity, with correlation coefficients (r^2^) between 0.990 and 0.999.

To evaluate the matrix effect in the samples, the signal suppression or enhancement (SSE) as a function of the slopes of the calibration curves obtained in methanol and in blank was obtained using the following formula:

SSE(%) = 100 × slope with matrix/slope without matrix.

SSE values above 100% indicate signal enhancement, while those below 100% indicate signal suppression. Therefore, values close to 100% indicate that there is no significant matrix effect. The results obtained indicated that there was signal suppression for OTA and AFs, which was more pronounced for OTA (52%), whereas no pronounced signal suppression/enhancement was observed for ENNs (73–84%). Therefore matrix-matched calibration curves were used for effective quantification of the samples.

The limit of detection (LOD) and limit of quantification (LOQ) were calculated by spiking a blank cricket sample with decreasing concentrations of the mycotoxins studied (from 0.1 to 500 μg/kg). The LODs were calculated taking into account the ratio of signal to background noise, which had to be equal to or greater than 3; for the calculation of the LOQs, the signal-to-noise ratio had to be equal to or greater than 10. The LODs and LOQs ranged from 0.03 to 0.3 µg/kg and from 0.1 to 1 µg/kg, respectively.

The recoveries were determined using cricket blanks. For this, cricket blank samples were spiked with each mycotoxin tested at a concentration of 100 × LOQ before and after QuEChERS extraction in triplicate. Intra-day precision was assessed with three determinations performed on the same day and inter-day precision was assessed with three determinations performed on non-consecutive days. Recoveries ranged from 64% to 119% and intra-day and inter-day precision were lower than 14% and 20%, respectively. These values were within the relative standard deviation set [30].

### 2.7. Heavy Metals Analysis

As, Cd, Hg, and Pb were analyzed in the insect samples and the resulting cakes obtained by SFE, according to a previous study [31].

A microwave accelerated reaction system (MARS, CEM, Vertex, Spain) was used for acid mineralization of the samples. For this purpose, 300 mg of sample was weighed into a Teflon digestion vessel, and then 4 mL of nitric acid (HNO_3_) at 14 M and 1 mL of hydrogen peroxide (H_2_O_2_) at 30% *v*/*v* were added. The samples were digested for 15 min at 800 W and 180 °C. After digestion, the samples were allowed to cool to room temperature, the digested samples were filtered through Whatman No. 1 filter paper, and the volume was made up with distilled water. Finally, inductively coupled plasma mass spectrometer equipment (ICP-MS, Agilent model 7900) was used for the determination. The method conditions were set as follows: carrier gas flow (1.07 L/min), Ar gas flow (15.0 L/min), reaction gas (He), RF power (1550 W), nebulizer pump speed (0.10 rps), and RF coincidence (1.80 V).

For As, Cd, and Pb, standard calibration curves from 0 to 1000 μg/L were used. A standard calibration curve from 0 to 100 μg/L was used for the quantification of Hg. The limit of detection (LOD) was calculated using the following formula: LOD = 3sB/a, where “3sB” is 3 times the standard deviation at zero concentration and “a” is the slope of the calibration curve. The LOD values obtained for As, Hg, Cd, and Pb were 0.012, 0.0015, 0.004, and 0.0015 μg/L, respectively.

### 2.8. Statistical Analysis

Statistical analysis was conducted on heavy metal content using a two-way ANOVA test with 95% significance (*p* < 0.05). Results were processed in GraphPad Prism 8.0.2 software (GraphPad Software, San Diego, CA, USA) and expressed as mean ± SD (standard deviation).

## 3. Results and Discussion

### 3.1. Occurrence of Mycotoxins in Insects

**Figure 1:**
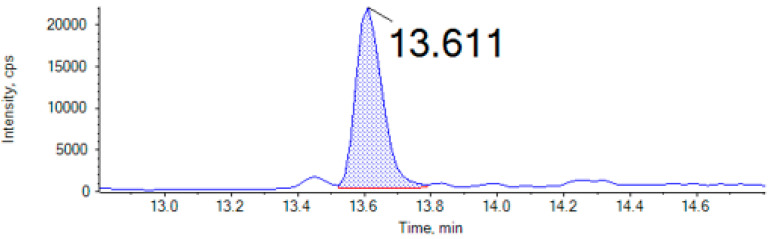
Chromatogram of cricket sample contaminated with Enniatin B (ENNB) (2.2 µg/kg) determined by UHPLC-MS/MS.

Three different types of mycotoxins were analyzed from all insect samples: AFs (AFB1, AFB2, AFG1, and AFG2), OTA, and ENNs (ENNA, ENNA1, ENNB, and ENNB1). Mycotoxin occurrence indicated that four samples of cricket, mealworm, buffalo worm, and locust were contaminated by at least one mycotoxin (Table 2). ENNs were detected with the highest frequency. They were present in all insect samples, with the exception of mealworms, with contamination levels ranging from 0.7 to 2.2 μg/kg. Figure 1 shows a chromatogram of a cricket sample contaminated with ENNB (2.2 µg/kg). ENNA was only present in crickets, and the rest of the ENNs (ENNA1, ENNB, and ENNB1) occurred in crickets, locusts, and buffalo worms. The levels detected in insects are lower than those reported by other authors for ENNB in cereal-based products (6–269 µg/kg) or fish from aquaculture (1.3–72.3 µg/kg) [32,33].

Regarding AFs, only AFG2 was detected at a level of 115.5 μg/kg in the locust sample (Table 2). In accordance with the regulations established on the presence of AFs in this type of food, locusts exceeded the limit of AFs by containing more than 4 μg/kg, which is the maximum permitted value set by the sum of aflatoxins B1, B2, G1 and G2 [34]. AFs occurrence was studied in dried EIs from Zambia (caterpillars and termites) by lateral flow immunochromatography. Average aflatoxin concentrations in termites *Macrotermes falciger* (Gerstacker) (24 µg/kg) exceeded regulatory limits established in Zambia (10 µg/kg). Moreover, when samples were subjected to poor storage conditions, AFs increased to unsafe levels in caterpillars. These authors also highlighted the need for proper storage of dried insects in order to mitigate AFs production [35].

**Figure 2:**
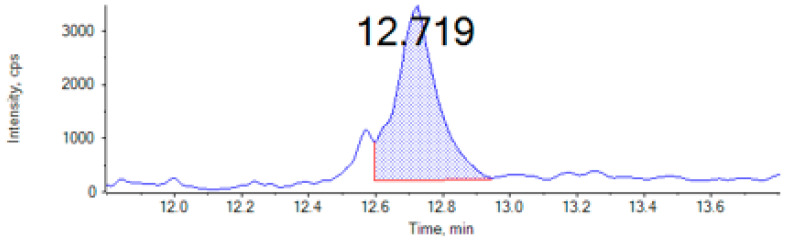
Chromatogram of mealworm sample contaminated with Ochratoxin A (OTA) (58.1 µg/kg) determined by UHPLC-MS/MS.

Finally, OTA was only detected at a level of 58.1 μg/kg in the mealworm sample (Figure 2). To the best of our knowledge, there is little information in the literature on OTA levels in insects. The majority of available studies have investigated the tolerance and excretion of OTA by insects, as detailed below. Regarding the occurrence of other mycotoxins, De Paepe et al. [36] evaluated the presence of the mycotoxins alternariol (AOH), alternaroyl methyl ether (AME), HT2 toxin, nicarbazin, nivalenol (NIV), roquefortine C, and zearalenone (ZEA)) by UHPLC-Q-Orbitrap™-HRMS in larval samples of mealworms (*Tenebrio molitor*), adult crickets (*Acheta domesticus*), and grasshoppers (*Locusta migratoria*). The insect samples were acquired from pet shops located in Belgium. Regarding the *T. molitor* samples, AOH was detected at a level < 15 μg/Kg, HT-2 at >15 μg/Kg; roquefortine C at a concentration < 30 μg/Kg, while nicarbazin was observed in all samples at concentrations > 100 μg/Kg. In *A. domesticus*, AOH was found at levels < 100 μg/Kg. Finally, in *L. migratoria*, nicarbazin (>100 μg/Kg) and NIV (3–6 μg/Kg) were detected. No AFs, OTA, or ENNs were analyzed by these authors, but the levels observed for other mycotoxins were in line with the concentrations determined in this work in these species of insects.

Contamination of insects with mycotoxins generally occurs during farming, when they are fed with feed that has already been contaminated, or as a result of poor handling and storage practices. The organism of these animals contains metabolic enzymes capable of degrading and excreting these mycotoxins, such as cytochrome P450, whose main function is to catalyze chemical reactions leading to the inactivation or elimination of these toxins [13]. Camenzuli et al. [37] discussed the OTA tolerance of *Alphitobius diaperinus* when exposed to concentrations ranging from 0.1 to 2.5 mg/kg for 10 and 14 days, respectively. It was found that approximately 100% of the OTA was excreted when feed was previously contaminated. During the study, the tolerance of *H. illucens* and *A. diaperinus* to feed artificially contaminated with a mixture of the mycotoxins AFB1, ZEN, DON, and OTA was tested at concentrations of 1, 10, and 25 times higher than the European maximum limits set in feed (0.02, 0.5, 5, and 0.1 mg/kg, respectively). It was also found that the insect response to a mixture of mycotoxins was no different than when exposed to feed contaminated with a single type of mycotoxin. Moreover, it has been demonstrated that species of *T. molitor* larvae fed on substrates contaminated with AFB1 or mycotoxin mixture (AFB1, OTA, ZEN, and DON) did not affect results in terms of weight gain and survival. More information is needed on the excretion capacity of mycotoxins or possible biotransformation into undetected or unknown chemical compounds [38].

The results for mycotoxin presence in insects’ defatted cakes obtained after SFE showed that OTA and AFG2 were detected at levels < LOQ after the SFE process, while emerging mycotoxins were detected at low levels, up to 1.3 µg/Kg in the case of ENNA in crickets and up to 0.8 µg/Kg for ENNA1, ENNB, and ENNB1 in crickets and locusts. Thus, mycotoxins could be reduced in defatted cakes after SFE technology. Several studies have reported the potential of technologies involving the application of high pressure, such as SFE and high hydrostatic pressure (HPP), for the inactivation of pathogenic microorganisms and mycotoxins [39]. For example, various authors have reported reduction rates ranging from 20 to 100% for mycotoxins such as aflatoxins, patulin, citrinin, and fusarium mycotoxins when HPP treatments between 250 and 600 MPa were applied to various food matrices for several minutes [40,41].

### 3.2. Occurrence of Heavy Metals in Insects

**Figure 3:**
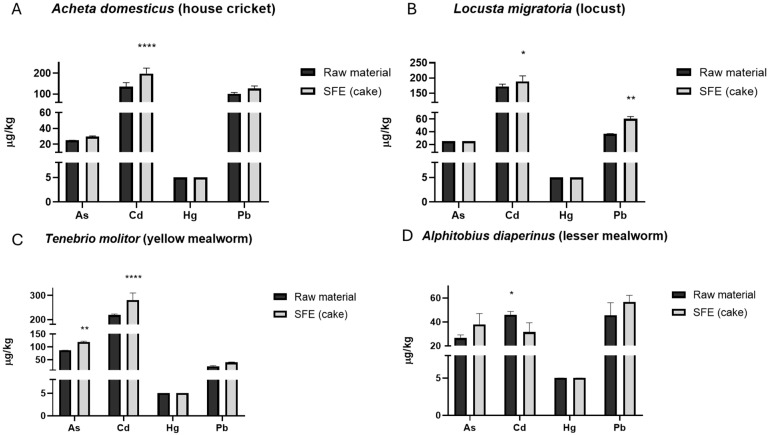
Heavy metal levels in edible insect samples: raw material vs. resulting cake obtained after supercritical fluid extraction. (**A**) *Acheta domesticus* (cricket) flour, (**B**) *Locusta migratoria* (locust), (**C**) *Tenebrio molitor* (mealworm) flour, and (**D**) *Alphitobius diaperinus* (buffalo worm). * = *p* < 0.05; ** = *p* <  0.01, **** = *p* < 0.0001 vs. control (raw material).

The presence of heavy metals (As, Cd, Hg, Pb) in the four insect species, as well as in the resulting cakes obtained after SFE extraction, was determined by ICP-MS. Table 3 shows the occurrence of heavy metals in the raw samples of crickets, mealworms, buffalo worms, and locusts, whereas Figure 3 shows heavy metal levels on the SFE cakes. A comparison between heavy metal levels in raw samples and SFE cakes is also shown in Figure 3.

For crickets, the amount of Cd and Pb quantified was 134.33 μg/kg and 100.33 μg/kg, respectively. Thus, the values set by Regulation (EU) 2022/188 (60 μg/kg for Cd and 50 μg/kg for Pb) were exceeded [42]. Lower values were found for As (25 μg/kg). In Locust, the level detected for Pb (36.7 μg/kg) was within the established limit, while Cd (172 μg/kg) exceeded the maximum levels set by Regulation (EU) 2021/1975 (70 μg/kg) [34]. As was detected at levels < 25 µg/kg. In the case of mealworms, the level of Pb (24 μg/kg) did not exceed the limit set, contrarily to Cd (219 μg/kg), with a maximum permitted of 100 μg/kg [43]. As was detected at a concentration of 86.7 μg/kg, but no maximum level has been still set for As in insects. Finally, in the case of buffalo worms, the concentrations of Pb (45.5 μg/kg) and Cd (46.05 μg/kg) were within the established limits [44], As occurred at the level of 26.5 μg/kg. Finally, it is important to note that in all insect species studies, Hg was the heavy metal detected at lower levels (<5 μg/kg).

Further, the evaluation of SFE cakes showed that heavy metals remain in the resulting solid after extraction (Figure 3), so practically no heavy metals are transferred to the extracted oils. Thus, the oils obtained could be safely incorporated into food.

As observed overall in Figure 3, in general, no significance was found when evaluating heavy metals in insect raw materials vs. SFE cakes, with the exception of Cd, where significantly (*p* < 0.05) higher amounts were found in crickets (134.33 vs. 198.5 μg/kg after SFE), locusts (172 vs. 189 μg/kg), and mealworms (219 vs. 281 μg/kg). For buffalo worms, Cd levels were significantly higher (*p* < 0.05) in insect raw material (46.05 μg/kg) compared to SFE cakes obtained after extraction (31.5 μg/kg), which should be noted. Additionally, Pb content was significantly higher in SFE cakes of locusts (36.70 vs. 60.5 μg/kg after SFE, *p* < 0.05), and the same trend was observed for As in mealworm (86.67 vs. 119 μg/kg, *p* < 0.05).

Poma et al. [14] evaluated metals (As, Cd, Co, Cr, Cu, Ni, Pb, Sn, Zn) in several species of EIs (greater wax moths, migratory locusts, mealworm beetles, buffalo worms). These authors reported Cd levels up to 60 μg/kg and Pb levels < 30 μg/kg. The Cd levels found in the present study were higher in grasshoppers and mealworms with concentrations of up to 219 μg/kg, but similar amounts were found in buffalo worms (46.05 μg/kg). In the case of Pb, the levels found (24–45.5 μg/kg) were slightly higher than those observed by these authors.

In another study, Kolakowski et al. [45] investigated the occurrence of heavy metals (As, Cd, Hg, Pb) in nineteen samples of crickets and silkworms. The incidences of As, Cd, Pb, and Hg were 100, 79, 58, and 74%, respectively. The detected concentrations ranged from 30 to 340 μg/kg for As, from 31 to 230 μg/kg for Cd, from 19 to 59 μg/kg for Pb, and from 0.94 to 28 μg/kg for Hg. In the present study, the levels of Cd (46–219 μg/kg) and Hg (<5 μg/kg) were within the range observed by these authors, but lower concentrations were found for As (<25–86.7 μg/kg) and higher amounts for Pb (24–100.3 μg/kg).

Other authors suggested that heavy metals could be bioaccumulated in the insect organism. In this sense, Vijver et al. [46] evaluated the adsorption of Cd, Pb, Cu, and Zn in *Tenebrio molitor* larvae and observed that after exposure to different types of soils with high concentrations of Cd and Pb, the larvae increased body concentrations of heavy metals.

These findings support the need for proper handling, processing, packaging, and storage conditions in insect farming. Some measures, such as controlling the presence of fungi, mycotoxins, and heavy metals in substrates intended for insect feeding and maintenance and appropriate environments (humidity, temperature, etc.) for insect breeding should be implemented [38].

## 4. Conclusions

Insects are nutritious and provide important benefits as a food source; however, they can accumulate hazardous chemicals such as mycotoxins and heavy metals. In terms of mycotoxins, only AFG2 and OTA were detected in the present study at levels above the legal limits in locusts and mealworms, respectively. The other mycotoxins analyzed were poorly detected or at low levels. Thus, the insects were not highly contaminated with mycotoxins. Regarding heavy metals, it was observed that in the four insect species studied, Cd was the heavy metal found in the highest concentration, with values up to 219 μg/kg, exceeding the limits established by the regulation, whereas Hg was detected at levels < 5 μg/kg. The results obtained after SFE extraction showed that heavy metals remained in the resulting SFE cakes, while mycotoxins were detected at negligible levels. Appropriate measures should be taken during insect farming, handling, processing, packaging, and storage to avoid the occurrence of hazardous chemicals.

## Figures and Tables

**Table 1:** Mass spectrometry and analytical parameters for method optimization for mycotoxins determination.

Mycotoxin	*t*_R_ (min)	DP	Precursor Ion (*m*/*z*)	Quantification Ion (Q)			Confirmation Ion (q)			SSE%	Recovery	LOQ µg/Kg	LOD µg/Kg
				CE	Product Ion (*m*/*z*)	CXP	CE	Precursor Ion (*m*/*z*)	CXP				
AFB1	8.1	106	313.1	33	285.2	16	91	128.1	10	66	114	1	0.3
AFB2	7.7	96	315.1	37	287.2	18	43	259.2	18	64	93	0.1	0.03
AFG1	7.5	86	329.1	39	243.1	14	59	200	12	63	119	0.1	0.03
AFG2	7.2	111	331.1	35	313.2	18	43	245.2	14	65	106	1	0.3
OTA	10.1	91	404	37	239	16	105	102	14	52	118	0.1	0.03
ENNA	12.6	106	699.4	43	210.1	12	47	228	18	84	64	0.1	0.03
ENNA1	12.4	96	685.4	46	210.1	8	49	228.2	20	76	67	0.1	0.03
ENNB	12	81	657.5	45	196.3	18	47	214.1	18	73	65	0.1	0.03
ENNB1	12.2	111	671.4	43	196	12	41	210	12	80	71	0.1	0.03

***t*_R_**: retention time, DP: declustering potential, CE: collision energy, CXP: collision cell exit potential, SSE %: signal suppression/enhancement. LOQ: limit of quantification, LOD: limit of detection, AFB1: Aflatoxin B1, AFB2: Aflatoxin B2, AFG1: Aflatoxin G1, AFG2: Aflatoxin G2, OTA: Ochratoxin A, ENNA: Enniatin A, ENNA1: Enniatin A1, ENNB: Enniatin B, ENNB1: Enniatin B1.

**Table 2:** Mycotoxins levels detected in insect samples in this study.

	Mycotoxins	ENNA (µg/kg)	ENNA1 (µg/kg)	ENNB (µg/kg)	ENNB1 (µg/kg)	AFG2 (µg/kg)	OTA (µg/kg)
Insect Samples							
**Cricket**		1.4 ± 0.02	1.4 ± 0.03	2.2 ± 0.26	1.7 ± 0.14	nd	nd
**Locust**		nd	0.7 ± 0.04	1.2 ± 0.01	0.8 ± 0.04	115.5 ± 1.7	nd
**Mealworm**		nd	nd	nd	nd	nd	58.1 ± 0.4
**Buffalo worm**		nd	0.9 ± 0.03	1.3 ± 0.03	1.0 ± 0.05	nd	nd

nd: not detected; AFG2: Aflatoxin G2; OTA: Ochratoxin A, ENNA: Enniatin A, ENNA1: Enniatin A1, ENNB: Enniatin B, ENNB1: Enniatin B1.

**Table 3:** Heavy metals occurrence (As, Cd, Hg, Pb) in raw insect samples.

Insects Species	As (µg/Kg)	Cd (µg/Kg)	Hg (µg/Kg)	Pb (µg/Kg)
***Cricket***	25.00 ± 0.1	134.33 ± 20	<5	100.33 ± 7
***Locust***	<25	172 ± 8	<5	36.70 ± 0.4
***Mealworm***	86.67 ± 0.6	219 ± 4.2	<5	24 ± 4
***Buffalo worm***	26.5 ± 2.6	46.05 ± 2.8	<5	45.50 ± 11

## Data Availability

The original contributions presented in the study are included in the article, further inquiries can be directed to the corresponding author.
